# Spinal arteriovenous fistulas supplied by branches of internal iliac artery: clinical features and endovascular treatment outcomes

**DOI:** 10.1007/s00701-026-06853-z

**Published:** 2026-04-01

**Authors:** Dong Hyun Yoo, Kang Min Kim, Young Dae Cho, Hyun-Seung Kang

**Affiliations:** 1 Department of Radiology, Seoul National University College of Medicine, 103 Daehak-ro, Jongno-gu, Seoul, 03080 Korea; 2 Department of Radiology, Seoul National University Hospital, 101 Daehak-ro, Jongno-gu, Seoul, 03080 Korea; 3 Department of Neurosurgery, Seoul National University College of Medicine, 103 Daehak-ro, Jongno-gu, Seoul, 03080 Korea; 4 Department of Neurosurgery, Seoul National University Hospital, 101 Daehak-ro, Jongno-gu, Seoul, 03080 Korea

**Keywords:** Spinal arteriovenous fistula, Internal iliac artery, Spinal angiography, Embolization, Endovascular

## Abstract

**Purpose:**

Branches of internal iliac artery (IIA) are under-recognized as potential feeders of spinal AVF and may be easily overlooked on spinal angiograms. This study aims to report clinical features and endovascular embolization outcomes in patients with spinal AVFs supplied by branches of IIA.

**Methods:**

We reviewed medical records of 13 patients treated at our institution for spinal AVFs supplied by branches of IIA, focusing on clinical characteristics and procedural outcomes. In each instance, endovascular embolization was first-line therapy.

**Results:**

Presenting symptoms included lower extremity weakness (n = 10), sensory impairment (n = 13), and sphincter dysfunction (n = 9). Spinal MRI regularly exposed perimedullary venous signal voids, although dilated draining veins of cauda equina showed prominence in eight patients only. On angiography, the precise location of the AVF was dural in nine and epidural in four patients, with fistula level ranging from L5 to S4. There was one instance of feeding vessel rupture, which was promptly controlled without sequelae. Ten patients (76.9%) experienced immediate AVF occlusions postembolization, but three lesions did recur. Single endovascular procedures ultimately resulted in stable occlusions for seven patients (53.8%). Together with subsequent surgical disconnections (n = 3), myelopathic symptoms abated in 10 patients overall.

**Conclusion:**

Angiography studies targeting suspected spinal AVFs should include IIA branches. In patients with spinal AVFs fed by branches of IIA, endovascular embolization represents a safe clinical option, offering 53.8% stable occlusion rate, and may be considered as initial choice of treatment in selected cases.

**Supplementary Information:**

The online version contains supplementary material available at 10.1007/s00701-026-06853-z.

## Introduction

Spinal vascular malformations are rare and encompass a number of lesions, according to various classification systems [31]. Overall, related clinical presentations range from acute hemorrhage and progressive myelopathy to compressive neuropathy, reflecting inherent angioarchitectural traits. Progressive myelopathy, in particular, typically results from the perimedullary venous drainage of arteriovenous fistulas (AVFs).

Currently, early diagnosis of spinal AVFs remains a challenge for various reasons. Their often small magnitudes and nonspecific neurologic symptoms, coupled with technical rigors exacted during thorough angiographic imaging of the spine, may delay detection and render recovery incomplete [18, 21, 32]. Lower lumbar or sacral AVFs are also frequently supplied by vascular offshoots of internal iliac artery (IIA), which are under-recognized as potential feeders and may be easily overlooked on spinal angiograms. Despite an abundance of previously published studies focused on dural AVFs of sacrum [1, 13, 19, 26], similarly situated variants (ie, epidural AVFs) are seldom addressed in the literature; and data reported for endovascular treatment of spinal AVFs are presently quite limited.

Herein, we used our single-institution archives to analyze patients with spinal AVFs supplied by branches of IIA, assessing clinical features and outcomes after endovascular embolization.

## Methods

This study was approved by the local ethics review board, having waived written informed consent due to its retrospective nature. No funding was received for conducting this study. We searched the neurointerventional database at our tertiary care center for first-line transarterial embolizations of spinal AVFs fed by IIA branches, retrieving all procedures conducted between December 2007 and December 2023. A review of medical records served to collect demographics, such as age and sex, and pertinent clinical data. We also recorded presenting symptoms, including motor, sensory, or urinary/defecation issues, tracking intervals between symptom onset and treatment as well. Initial spinal cord dysfunction was assessed using modified Aminoff-Logue scale (mALS) [33].

Individual magnetic resonance imaging (MRI) sequences (1.5 T or 3.0 T) of the spine obtained at baseline were likewise subject to review, checking T2-weighted images (T2WI) for extent of hyperintense signaling (a marker of myelopathy) and identifying flow voids in dilated perimedullary veins. We additionally looked for dilated veins amidst cauda equina at filum terminale level, shown as either flow voids on sagittal T2WI or as enhancing curvilinear structures on contrast-enhanced T1-weighted images (CE T1WI), if available. Designations applied in grading such features were as follows: prominent (unmistakably tortuous and dilated draining vein); subtle (single, mildly curvilinear structure, not readily visible among nerve roots); or none. Analysis of digital subtraction angiography (DSA) images and 3-dimensional rotational angiography (3DRA) data helped to more precisely categorize each fistula by type (dural, epidural, or intradural) and level, while also delineating respective feeder arteries and venous drainage.

Finally, we examined procedural reports to determine arterial feeders targeted for embolization, embolic materials used, and related complications. Immediate radiologic outcomes were of interest, with particular attention to complete penetration of embolic material beyond shunt points. Clinical outcomes and follow-up documentation yielded data on symptomatic relief, MRI results, and further endovascular or surgical intervention needed.

## Results

### Study population and clinical presentations

**Fig. 1:**
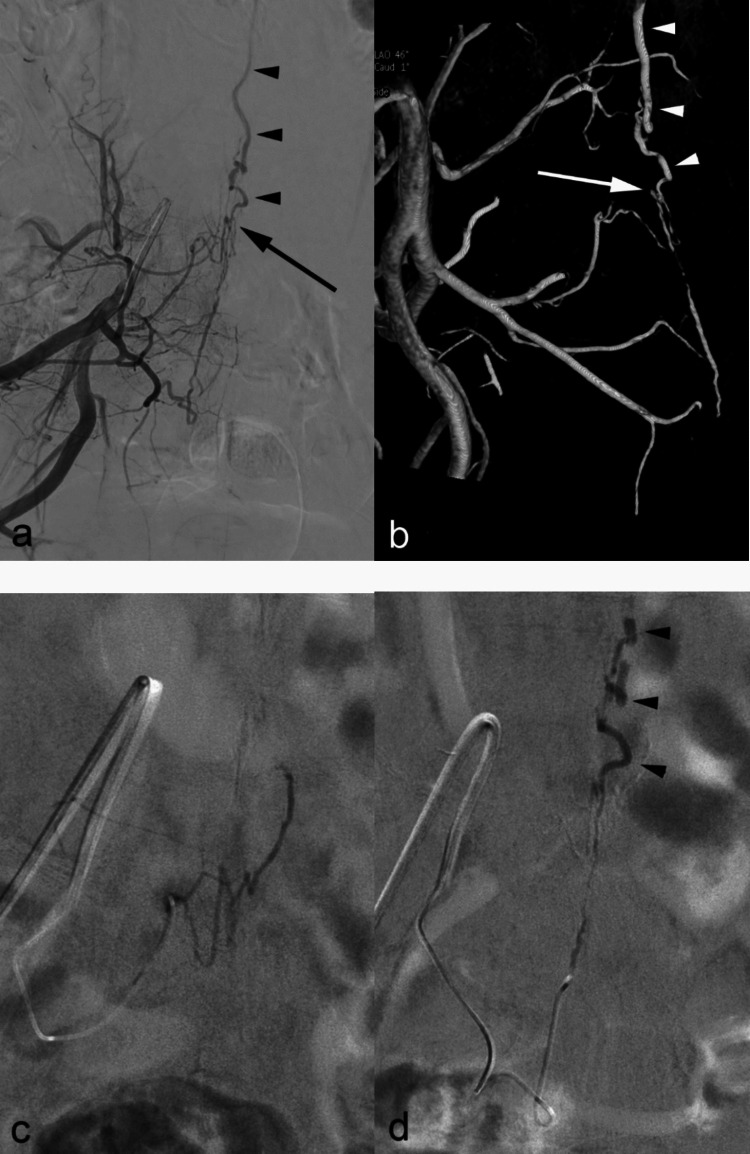
Case No. 5. (**a**) Right internal iliac DSA and (**b**) volume rendering image shows dural AVF at L5 level supplied by lateral sacral artery branch of S1 to S3. Shunt point in arrow and dilated filum terminale vein in arrowheads. (**c**) Glue mixture injection under local anesthesia from right S1 feeder artery failed to cast the shunt point. (**d**) Glue mixture injection from right S3 feeder artery successfully penetrated into draining vein (arrowheads)

**Fig. 2:**
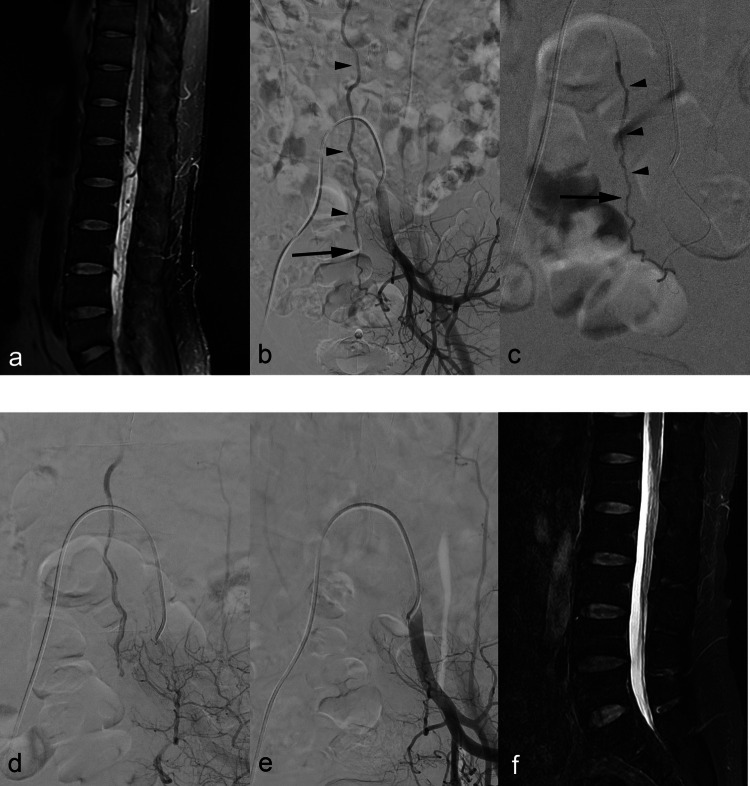
Case No. 7. (**a**) Sagittal T2 weighted MRI of lumbar spine shows dilated perimedullary veins and filum terminale veins among cauda equine, as well as mild spinal cord myelopathy. (**b**) Left internal iliac DSA shows dural AVF at L5 level, mainly supplied by lateral sacral artery branch of S4. Shunt point in arrow and dilated filum terminale vein in arrowheads. (**c**) Glue injection under general anesthesia from left S4 feeder artery. Successful penetration across the shunt point (arrow) to draining vein (arrowheads). (**d**) Post-embolization left internal iliac DSA shows sluggish residual shunt supplied from iliolumbar artery. Glue cast is seen as filling defect within the dilated filum terminale vein. (**e**) Follow up DSA 2 weeks later. Completely disappeared fistula and early draining veins. (**f**) 3 months follow up MRI reveals disappeared dilated perimedullary/filum terminale veins and cord myelopathy; patients remained symptom free during further 3 years of follow up

**Fig. 3:**
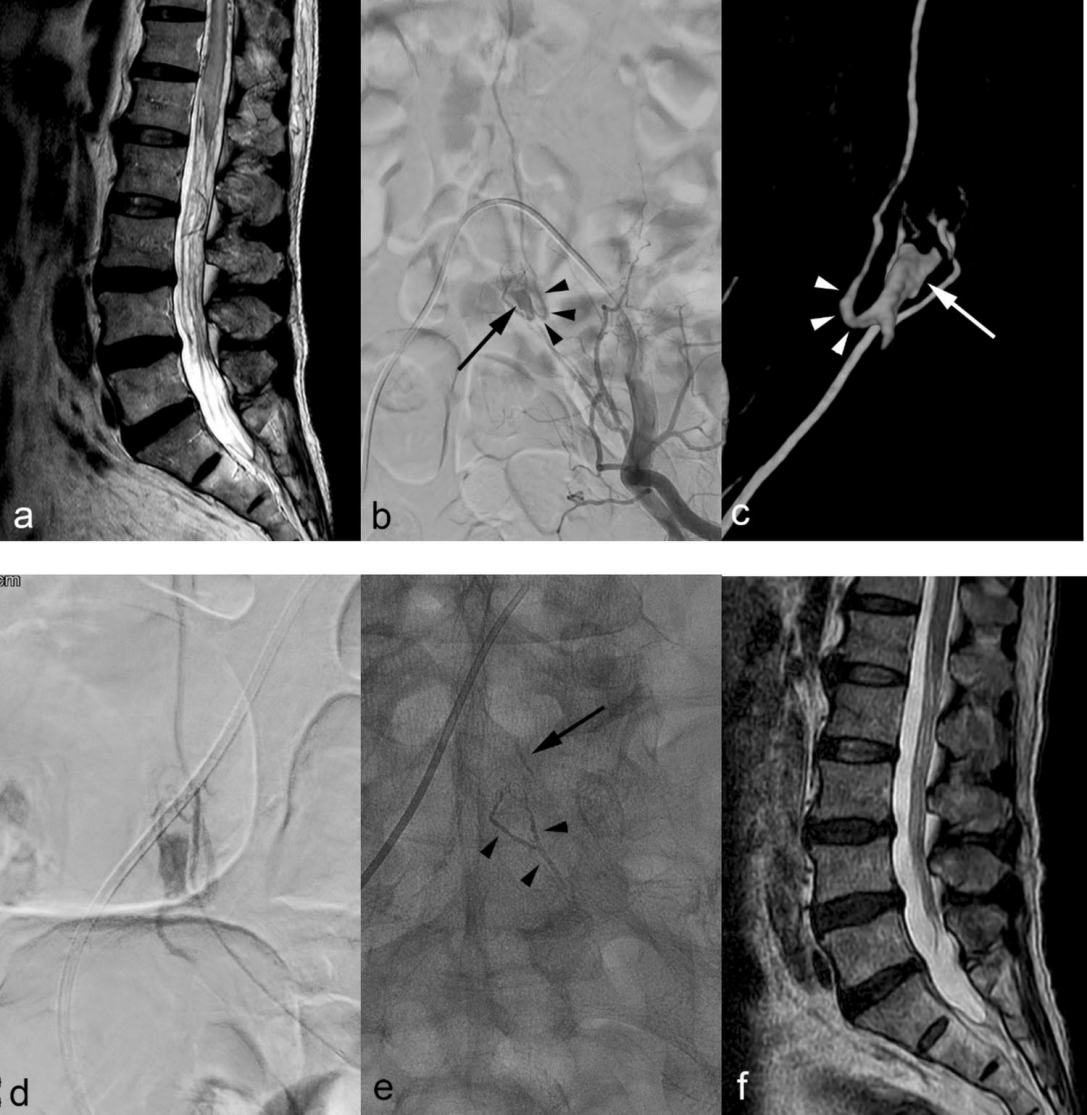
Case No. 13. (**a**) Sagittal T2 weighted MRI of lumbar spine shows dilated perimedullary veins and filum terminale veins among cauda equine, as well as spinal cord myelopathy. (**b**) Left internal iliac DSA and (**c**) volume rendering image posterior view shows epidural AVF at S1 level; bifurcated left S2 feeder artery supplies arterialized epidural pouch (arrow), which reflux to perimedullary veins via intradural radicular vein (arrowheads). (**d**) Right oblique view of selective DSA from a feeder artery. Glue was injected from this location under local anethesia. (**e**) Post-embolization spot image. Although glue cast is not visible in the epidural pouch, bifurcated feeder artery (arrowheads) and draining radicular vein (arrow) is occupied by glue cast. Immediate post-embolization DSA demonstrated no visualization of residual dural AVF (not shown). (**f**) 6 months follow up MRI reveals disappeared dilated perimedullary veins and cord myelopathy; patients improved clinically

**Fig. 4:**
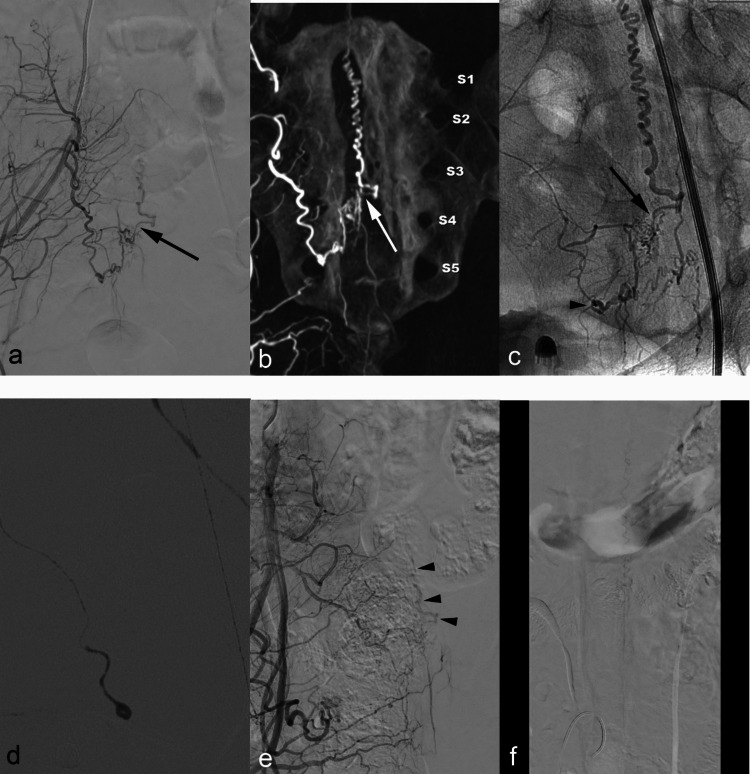
Case No. 12. (**a**) Right internal iliac DSA (A) and (**b**) MIP image. Dural AVF at S4 level is supplied by right lateral sacral artery branch of S3 to S5 (shunt point in arrow). (**c**) Left oblique view of selective S5 angiography shows network of arterial feeder, shunt point (arrow) and tortuous filum terminale vein (microcatheter tip in arrowheads). (**d**) Premature reflux of glue injection (under general anesthesia) failed to penetrate shunt point. Immediate post-embolization DSA demonstrated no visualization of residual dural AVF (not shown), but patient’s symptom persisted. (**e**) DSA 6 months later. Note the slow filling of dural AVF through fine network of arterial feeders (draining vein in arrowheads). (**f**) Delayed phase of right internal iliac DSA at lumbar level shows venous drainage into tortuous and dilated perimedullary veins

Between December 2007 and December 2023, 13 patients (men, 8; women, 5) with spinal AVFs supplied by IIA branches underwent endovascular treatments at our institution (Table 1). Representative cases are depicted in Figs. 1, 2, 3, 4. Mean age was 51.8 years (range, 31–73 years). Overall, presenting symptoms included lower extremity weakness (*n* = 10), sensory impairment (*n* = 13), and sphincter dysfunction (*n* = 9). Median mALS was 4 (IQR, 3). Time from symptom onset to diagnosis by DSA ranged from 10 days to 10 years (mean, 18.8 months). In three instances, there were concomitant perimedullary AVFs at conus medullaris (Case No.2 and 8) or filum terminale (Case No. 6). Case No. 3 exhibited coexistent spinal disease, harboring a tethered cord with lipomeningomyelocele.

**Table 1:** Summary of clinical findings

Case no	Age	Sex	Onset to diagnosis	Symptoms				
				Motor	Sensory	Urinary	Bowel	mAL
1	33	F	1 month	+	+	-	-	1
2	34	M	3 months	-	+	+	+	5
3	41	F	10 years	-	+	+	+	3
4	72	F	13 months	+	+	-	+	4
5	31	M	2 months	+	+	-	-	1
6	58	M	19 months	+	+	+	-	7
7	53	M	3 months	-	+	+	+	4
8	37	M	10 days	+	+	-	-	5
9	67	F	2 months	+	+	-	+	3
10	67	M	9 months	+	+	+	+	8
11	54	M	12 months	+	+	+	+	4
12	53	F	4 years	+	+	+	+	7
13	73	M	12 months	+	+	+	-	3

*mAL* modified Aminoff-Logue scale

### Diagnostic imaging

Preoperative spinal MRI studies revealed T2 hyperintensity of the spinal cord (signifying myelopathy) in 10 patients, and regularly present signal voids attested to perimedullary venous engorgement in all patients. The latter largely involved mid-to-lower thoracic spine but did reach as far as cervical spine level (C6 in Case No. 10). On T2WI, dilated veins of the cauda equina qualified as prominent in eight patients (61.5%) and subtle in two patients (15.4%), with three patients (23.1%) demonstrating none. CE T1WI were available for six patients, and additional value to T2WI was present in a sole case (Case No. 4), where a single, subtly enhanced strand of dilated vein was detected (Table 2).

**Table 2:** Summary of spinal MRI and angiogram findings

Case no	Myelopathy extent	PM flow void	Dilated vein among cauda equina (T2WI/CE T1WI)^a^	Fistula location	Fistula level	Feeders
1	none	L1 -	+ +/NA	dural	S2	Lt S3
2	T12 -	L1 -	+ +/+ +	dural	S1	Rt S2
3	L1 -	T12 -	+ +/+ +	epidural	S1	Both L5
4	T8 -	T8 – L1	-/+	epidural	S1	Rt S1
5	none	T7 -	+ +/NA	dural	L5	Rt S1-3, Lt L5
6	T6 -	T6 -	+/NA	dural	S3	Rt S4
7	none	T5 -	+ +/NA	dural	S1	Lt S4
8	T9 -	T3 -	+ +/+ +	dural	S1	Lt S1-5
9	T10 -	T6 -	+ +/+ +	epidural	L5	Both L5^b^
10	T6 -	C6 -	+ +/NA	dural	L5	Rt L5
11	T7 -	T9 – L1	-/NA	dural	L5	Rt S1
12	T11 -	T8—L2	-/-	dural	S4	Rt S3-5
13	T8 -	T5 -	+/NA	epidural	S1	Rt S2

*CE* Contrast enhanced, *NA* not available, *PM* Perimedullary

^a^ + + : prominent, + : subtle, -: none

^b^ Right L5 artery originated from median sacral artery while left L5 artery originated from left internal iliac artery

The AVFs under study occupied dural (n = 9) or epidural (n = 4) locations exclusively. There were no intradural lesions supplied by IIA branches, involving filum terminale for instance. These fistulas occupied L5 level in four patients, S1 level in six patients, and levels below S2 in three patients. The IIA furnished unilateral feeder arteries in 10 patients, with three lesions receiving bilateral IIA branches or added supply from median sacral artery (Table 2). Two AVFs were missed on earlier spinal angiograms. In Case No. 6, angiography performed elsewhere (18 months prior) had omitted IIA. Another epidural AVF (Case No. 4) failed to clearly materialize during right common iliac angiography, hindered by insufficient contrast injection into IIA and inordinate rectal retention of barium enema contents. Repeated testing 5 months later finally achieved exposure through selective right IIA angiography.

### Endovascular procedure and follow-up

The outcome of endovascular procedure is summarized on Table 3. The decision to perform embolization under general anesthesia was based on the operator’s preference, also considering the patient’s ability to cooperate. As a result, 6 patients (Case No. 1, 2, 4, 5, 6 and 13) were treated under local anesthesia, while general anesthesia was applied in 7 patients. All endovascular treatments entailed navigating a microcatheter reasonably close to each AVF under fluoroscopic and roadmap guidance. For embolization purposes, we used a glue mixture (n-butyl cyanoacrylate [NBCA] and lipiodol) at 20–33% concentration, depending on feeder size and distance from microcatheter tip to shunt point. This material was applied to single feeders in 10 patients, selecting two separate feeders for embolization in the three remaining cases (Case No. 3, 5, and 10) (Fig. 1). On one occasion (Case No. 3), feeding vessel rupture occurred in the course of navigation but was promptly controlled by injected glue, conferring no adverse neurologic effects. Our procedures were otherwise free of immediate complications or delayed adverse effects.

**Table 3:** Summary of endovascular treatment and follow up

Case no	Immediate result	Glue vein penetration	Anesthesia	Clinical course	f/u time	Recovery
1	Complete	No	L/A	Stable occlusion	11 yr	Good
2	Complete	Yes	L/A	Recur → 2nd embo(fail)	14 yr	Partial
3	Complete	No	G/A	Recur → 2nd embo(fail) → surgery	7 yr	Good
4	Complete	Yes	L/A	Stable occlusion	9 mo	Partial
5	Complete	Yes	L/A	Stable occlusion	25 mo	Good
6	Complete	No	L/A	Stable occlusion	8 mo	No
7	Incomplete	Yes	G/A	Stable occlusion	39 mo	Complete
8	Incomplete	No	G/A	f/u loss	f/u loss	n/a
9	Complete	Yes	G/A	Stable occlusion	12 mo	Good
10	Complete	Yes	G/A	f/u loss	f/u loss	n/a
11	Incomplete	No	G/A	Surgery	3 mo	Complete
12	Complete	No	G/A	Recur → surgery	9 mo	Partial
13	Complete	Yes	L/A	Stable occlusion	6 mo	Partial

*G/A* General anesthesia, *L/A* Local anesthesia

In 10 patients (76.9%), disappearance of AVF was achieved on immediate postoperative angiograms of IIA (bilaterally) and median sacral artery. However, injected glue had traversed beyond shunt points to permeate draining veins in seven patients only. Although the AVF of Case No. 7 remained visible postoperatively, its large-caliber draining vein had been penetrated by glue mixture. Complete AVF occlusion was confirmed 2 weeks later during repeat angiography (Fig. 2). Eventually, stable occlusions prevailed in seven patients (53.8%) following single endovascular sessions, all corroborated by follow-up DSA findings, symptomatic improvement, and/or spinal MRI sequences devoid of venous engorgement. Surgery was successfully performed on one occasion (Case No. 11) where endovascular means failed to confer AVF occlusion on first attempt.

There were three instances of recurrence, one of which eventuated in immediate successful surgical disconnection (Case No. 12). In the other two, second embolization attempts failed, finding it difficult to superselect feeding arteries suitable for injection. One of these patients remained clinically stable, requiring no further treatment. The second underwent successful surgical disconnection.

Two patients were lost to follow-up, and a third abandoned outpatient monitoring 3 months after surgical disconnection, having completely recovered. All but one of the remaining 10 patients showed partial-to-complete, sustained resolution of symptoms during follow-up periods that ranged from 6 months to 14 years (mean, 49.1 months).

The concomitant intradural AVFs we encountered are clinically detailed in *Supplementary Materials*.

## Discussion

Spinal AVFs are supplied by segmental arteries of the spine, extending from cervical to sacral level. The posterior trunk of IIA gives rise to iliolumbar artery supplying L5 level and lateral sacral artery giving off branches to sacral segments. Spinal AVFs at L5-S5 levels are thus invariably supported by branches of IIA, occasionally in combination with feeders from median sacral artery. Herein, we have detailed 13 patients treated endovascularly for spinal AVFs supplied by IIA branches. This group accounted for 11.9% (13/109) of all patients with spinal vascular malformations who opted for endovascular treatments at our institution during the stated study period.

Clinical presentations of spinal AVFs are seldom specific to corresponding levels of arterial supply. Myelopathic symptoms are direct consequences of heightened venous pressure and impaired venous drainage, stemming from arterial shunting into perimedullary venous plexus [17, 22]. The severity and extent of venous congestion mirrors the overall distribution and patency of radiculomedullary and bridging veins connecting intra- and extradural venous systems, of which individual capacities vary considerably [12, 20]. Hence, thorough spinal angiography studies are essential in patients with clinically suspected spinal AVFs, ensuring that all segmental arteries from craniocervical junction (external carotid and vertebral arteries) to sacral level (IIA and median sacral artery) are included. Omission of IIA from diagnostic angiography done elsewhere created an 18-month delay for one of our study subjects; and delays in discovery are apt to worsen clinical outcomes [18]. In addition, it is crucial to fully opacify potential feeding arteries on angiogram of IIA. Spinal AVFs are well visualized from proximal IIA injection in most patients with appropriate contrast volume. However, more distal catheterization to IIA posterior trunk should be pursued if visceral branches or motion artifact obscure iliolumbar and lateral sacral artery.

For spinal AVFs of sacrum to impact perimedullary venous plexus, they must be directed craniad via filum terminale vein (FTV) [4, 10], producing detectable FTV engorgement alongside cauda equina on spinal MRI. Brinjikji et al. have reported high positive (94.9%) and negative (95.1%) predictive values for dilated FTV visibility on sagittal T2WI and/or CE T1WI of deep lumbar or sacral fistulas [5]. If venous dilatation and tortuosity are subtle, however, such changes may be obscured on T2WI in the midst of cauda equina. Present study findings indicate only eight patients (61.5%) with FTV prominence on T2WI. In three patients (23.1%) who lacked visible veins, CE T1WI obtained for two of them either failed at exposing veins or showed slight alterations requiring high degrees of suspicion by reviewers. Ultimately, engorged veins identifiable at filum terminale level make IIA angiography a clear priority over technically demanding and time-consuming thoracolumbar spinal angiography, thus avoiding unnecessary radiation and contrast use. Still, their absence on MRI does not exclude possible feeders from IIA branches.

Dural locations (9/13, 69.2%) were most common in our AVF series. Indeed, other case reports and patient series have indicated that dural-type sacral AVFs figure most prominently among AVF variants [1, 4, 13, 19, 24, 26, 28–30]. Epidural AVFs (4/13, 30.8%) emerged as the second most prevalent type in our series, their hallmark being a dilated epidural pouch [6] (Fig. 3). Cauda equina syndrome may result from pouch-related mass effect, or myelopathy may ensue due pouch drainage and subsequent perimedullary venous reflux [7]. One epidural AVF in our series occurred concurrently with lipomenigomyelocele, which shares facets of a case previously published by Giordan et al. [14]. The authors have suggested a pathophysiologic link between spinal dysraphism and spinal AVF, both conditions resulting from defective embryologic layer migration during neural tube formation. Although reports of intradural located AVFs of filum terminale with lateral sacral artery supply have already surfaced [11, 15, 23], we have yet to encounter any lesions of this sort.

What we unexpectedly determined was a high prevalence (3/13, 23.1%) of concomitant intradural AVFs at conus medullaris and filum terminale. This rare disease combination has been previously described, but it is uncertain whether their coexistence is incidental or related [16, 27]. One hypothesis is that AVF-induced venous hypertension incites local thrombosis, spawning another lesion at a distant level. Persistence of patient symptoms despite successful treatment should therefore raise the question of an additional lingering lesion. An equally remarkable case in our series involved spontaneous regression of a conus perimedullary AVF (Case No. 8, *see Supplementary material*). Such an event is considered extremely rare and supports the dynamic nature of AVF lesions [25]. It is unclear whether its recent disappearance sufficiently altered the hemodynamic of sacral dural AVF drainage to prompt sudden worsening of patient’s symptoms, or earlier attempts at AVF closure enabled a functional 20-year period free of interventions and symptom aggravation is solely ascribed to hemodynamic change of sacral dural AVF.

Permanent obliteration of shunt points, by way of surgical disconnection or liquid embolic casting, is imperative for curative treatment of spinal AVFs. While offering near-total cure rates, direct surgical interventions are highly invasive and require prolonged recovery periods. Endovascular embolization by glue injection is comparatively less intrusive and is backed by favorable reported success rates. Another advantage of embolization is possible treatment without the use of general anesthesia, if the operator feels comfortable at favorable vascular access to the lesion and stability of the patient. In addition, embolization may be attempted at the same session as diagnostic angiography, mitigating the risk of delayed treatment. In our series, among the 6 embolization cases performed under local anesthesia, 3 patients (Case No. 1, 5, and 6) underwent embolization at the same session as diagnostic angiography resulting in complete occlusion with favorable clinical outcome. Although local anesthesia raises concern of movement artifact and deterioration of angiographic image quality, 3DRA under breath hold usually resulted in optimal image at lumbosacral area, by which precise angioarchitecture could be defined. Moreover, after distal selection of arterial feeder with microcatheter, maneuvering C-arm could achieve suitable projection angle with least motion interference on subsequent glue injection. Therefore, in patients with full cooperation and stable condition, there was no endovascular procedural compromise due to patient motion even under local anesthesia. In our series, embolization under local anesthesia showed higher immediate complete occlusion rate (6/6, 100% vs. 4/7, 57.1%) and stable occlusion on follow up (5/6, 83.3% vs. 2/7, 28.6%). However, it might be that case with less complex arterial feeder have been applied local anesthesia. Also, due to small number of cases and varying follow up period (including follow up loss), implications of this finding is limited.

The most critical step in embolization is the penetration of glue beyond shunt points to the foot of draining veins. Even in AVFs non-visualized postembolization, failure to completely occlude shunt points may encourage recurrence via fine collateral feeders. This was the apparent flaw for two patients (Case No. 3 and 12) in our series. Prompt follow-up imaging (DSA or MRI) and careful monitoring of symptoms are indicated under these circumstances. Then again, even though residual shunt flow persists on postembolization angiography, partial casting of draining veins (Case No. 7, Fig. 2) using a glue mixture may promote eventual shunt occlusion, owing to the high thrombogenicity of NBCA.

We have found that embolization across branches of IIA results in moderate therapeutic success, frequently affording stable occlusions (7/13, 53.5%) over time after single endovascular procedures. Although Case No. 10 had abandoned outpatient visits, durable occlusion was highly probable, knowing the glue mixture impacted all AVF components (arterial feeder, shunt point, and draining vein). As a rule, lesions with single-level feeders are effectively obliterated through superselection and straightforward glue mixture injection. Small feeders originating at multiple levels and forming supply networks reduce the proximity of microcatheter navigation to shunt points, increasing chances of incomplete occlusion (Fig. 4). Consequently, the applied glue does not penetrate beyond shunt points at times (Case No. 8 and 12). Ethylene vinyl alcohol (EVOH) copolymer-based liquid embolic agents (Onyx [Medtronic, Minneapolis, MN, USA] and PHIL [Terumo Neuro, Aliso Viejo, CA, USA]) are advantageous if prolonged and controlled injections are needed. Although their efficacies and long-term outcomes in treating spinal AVFs remain in question [2, 3], recent studies reported safety and effectiveness of these agents using novel approaches such as pressure cooker technique or balloon pressure technique [8, 9]. We were not able to apply these materials or techniques to our case series since local regulation restricted use of EVOH in spinal vascular lesions.

In terms of embolization safety, spinal AVFs supplied by IIA branches seem less prone to procedural complications. This may be explained on the following basis: 1) spinal arteries seldom arise in this vicinity, so the potential for inadvertent spinal cord infarction is low, and 2) there is less chance of glue migration to an intact perimedullary venous plexus, because the distance from shunt point to spinal cord is rather long [13]. Our only procedural complication was vessel rupture during navigation, which was controlled without any sequelae.

There are certain limitations of the present study to acknowledge. Given its retrospective nature, a lack of uniformity in clinical evaluations and procedural protocols was unavoidable. For example, the presenting symptoms of patients were not systematically assessed; and follow-up intervals after procedures varied among patients, with two patients lost to follow-up immediately post-treatment. In most instances, postprocedural disability was not documented fully on medical record to determine mALS. Together with reason that mASL did not reflect improvement on pain and sensory symptom, clinical recovery was assessed partly relying on subjective patient-reported relief of symptom. On occasion, concomitant AVFs situated at separate levels made it difficult to gauge treatment efficacy. We utilized various microcatheter types and glue concentrations throughout the study. Although EVOH could be effective for lesions that are difficult to navigate near the shunt point, it was not available due to local regulations. Finally, the small number of cases involved precluded any meaningful statistical analysis.

## Conclusion

Spinal AVFs of lower lumbar and sacral areas are supplied by IIA branches. Because related clinical presentations are not specific to levels at which lesions or feeders are situated, spinal angiography compelled by clinical suspicions should include IIA evaluations with full opacification of iliolumbar and lateral sacral artery. Endovascular embolization may be viewed as a safe option in this setting, capable of 53.8% stable occlusion rate. Endovascular treatment could be a preferred first-line option in selected cases allowing same-session diagnostic angiography and embolization under local anesthesia; should this fail, more invasive but definite surgical disconnection may ensue.

## Supplementary Information

Below is the link to the electronic supplementary material.

Supplementary file1 (DOCX 16 KB)

## Data Availability

No datasets were generated or analysed during the current study.
