# Supplementary material for: Spinal arteriovenous fistulas supplied by branches of internal iliac artery: clinical features and endovascular treatment outcomes
Source: Acta Neurochir (Wien). 2026 Apr 1;168(1):92. doi: 10.1007/s00701-026-06853-z (PMC13046627; doi:10.1007/s00701-026-06853-z)
Supplement: Supplementary file 1 — Supplementary file1 (DOCX 16 KB) [file 701_2026_6853_MOESM1_ESM.docx]

***Clinical course of the concomitant intradural AVF***

Case No. 2 presented with a concomitant perimedullary AVF at conus medullaris, supplied by anterior spinal artery from right T11 artery. The main source of symptomology being unclear, we planned to surgically disconnect this perimedullary lesion following embolization of a dural AVF at sacrum. However, residual perimedullary AVF persisted, despite two open surgical interventions. Also, some residual of the sacral AVF shunt was detectable 2 years after embolization and proved refractory our second embolization attempt. Nevertheless, this patient remained clinically stable for 14 years.

Case No. 8 had presented 20 years earlier with a concomitant perimedullary AVFs at conus medullaris, supplied by anterior spinal artery from right T10 and posterior spinal artery from left L3 artery. This lesion was considered the main source of myelopathy, warranting an embolization procedure that met with incomplete occlusion. The patient was then lost to follow-up, ignoring recommended surgical excision, only to present again after a 20-year hiatus with acutely worsened symptoms. Subsequent DSA showed spontaneous regression of the perimedullary AVF, with sacral AVF persistence. Surgical disconnection was offered once more following incomplete embolization, but the patient was lost to follow-up for a second time.

An AVF at filum terminale had gone undetected in Case No. 6 during diagnosis and embolization of a dural AVF at sacrum. Persistent symptoms in conjunction with diminished but persistent venous engorgement on MRI led to follow-up angiography 3 months later. This disclosed the filum terminale AVF, marked by marginal sluggish shunting and a filum terminale arterial supply from left T11 artery. Surgical treatment was waived, given little expected clinical benefit.
